# Assessment of Oral Health Status and Communication Barriers in Hearing- and Speech-Impaired Children in Jeddah City

**DOI:** 10.7759/cureus.23277

**Published:** 2022-03-17

**Authors:** Yagoub Alyami, Rakan N Alamri, Mohammad A Abdulsamad, Omar H Alsharabi, Muath M Hakami, Majdi A Alsheekh, Hany O Zamka, Mohammed A Alhijaili, Khalid A Alharbi, Rotana M Abulaban

**Affiliations:** 1 Oral and Maxillofacial Surgery, Vision Colleges for Dentistry and Nursing, Jeddah, SAU; 2 Dental Intern, Vision Colleges for Dentistry and Nursing, Jeddah, SAU; 3 Department of Oral Diagnosis, King Abdul-Aziz University Faculty of Dentistry, Jeddah, SAU

**Keywords:** poor oral hygiene, speech-impaired, oral health status, hearing, children

## Abstract

Background: Hearing- and speech-impaired people form a significant part of society. Literature reveals that these people have compromised oral health conditions as compared to people with decreased levels of oral health alertness and communication barriers. The aim of the study was to assess the oral health status in hearing- and speech-impaired children in Jeddah city.

Methodology: One hundred sixteen children aged five to 16 years of either gender with hearing and speech impairment were selected. Parameters recorded were dental caries, gingival diseases, and malocclusion. The fluorosis status was also recorded.

Results: The total number of children in the age group five to seven years was 16, eight to 10 years was 25, 11 to 13 years was 30 and 14 to 16 years was 45. Of the 116 children, there were 60 males and 56 females. Among the 116 children, 25 had decayed, 12 had missing and 30 had filled teeth. Malocclusion was found to be class II div I in 20, class II div II in 11, class III in five, spacing in 17, and rotation in 10. It was found that 32 had mild fluorosis and 74 had gingivitis. Method of communication was sign language in 62%, lip-reading in 20% and hearing aids in 18% of the population.

Conclusion: Special care needs to be taken in children with hearing and speech disabilities together with poor oral hygiene status. A careful oral examination is needed in these children.

## Introduction

Children are prone to develop a variety of dental diseases. Among them, dental caries, gingivitis, and periodontitis are common [1]. The increase in the prevalence of dental diseases among children is due to poor dietary habits, frequent sugar intake, and prolonged bottle feeding [2].

Children with hearing impairment are at high risk of developing dental diseases. When a person is not able to hear at 60 decibels (dB) or more, it is defined as hearing impairment. It is one of the most important and common hearing disabilities. In children, both speech and hearing disabilities are of great concern as they hamper growth and development. The prevalence was found to be 1/1000 live childbirth. It is estimated that the prevalence accounts for 0.5% of hearing and speech impairments [3,4].

The personal, as well as the overall health of children, is affected. It in turn affects the quality of life. Children face difficulty in communicating with their parents and others [5]. It has been shown in studies that the communication barrier results in mental suffering, abuse related to emotional and physical factors, practical difficulties, and poor social relationships [6].

Dental caries is a microbial disease of calcified structure categorized by the destruction of organic and dissolution of the inorganic component of the tooth. It is an irreversible phenomenon. Gingivitis is defined as inflammation of the gingiva [7,8]. Children with special needs have poor oral hygiene owing to disability of sensory, motor, and intellectual function and communication barriers. Patients with impaired hearing suffer difficulty in communication [9]. Considering this, the current study is aimed to assess oral health status in hearing- and speech-impaired children in Jeddah city.

## Materials and methods

This cross-sectional and observational study was done between the period 2019-2021. A total of 116 children aged five to 16 years were enrolled for the present research. All enrolled children had hearing and speech impairment. Ethical approval was given by Vision Colleges for Dentistry and Nursing Jeddah, Kingdom of Saudi Arabia, with approval number 19-02/05, and written consent for the enrollment of children with special needs was taken from respective parents.

Demographic data of each subject was recorded in the case history sheet. The children were observed under artificial light seated comfortably on an ordinary chair. A thorough oral evaluation was carried out using the mouth, mirror, and probe. Parameters recorded were dental caries, gingival diseases and malocclusion. The fluorosis status was also recorded. 

The data were analyzed using the Statistical Package for the Social Sciences (SPSS) version 19.0 software (IBM Corp., Armonk, NY, USA). Chi-square test was used to find the association between the gender, age group, and oral health parameters. 

## Results

The number of subjects in age groups five to seven years, eight to 10 years, 11 to 13 years and 14 to 16 years were 16, 25, 30 and 45 respectively. Out of 116 children, there were 60 males and 56 females. We observed a non-significant difference between males and females (P=0.210) (Table 1).

**Table 1:** Basic sample characters

Age group (years)	Number
5-7	16
8-10	25
11-13	30
14-16	45
Gender	
Male	60
Female	56

Among 116 children, 25 had decayed, 12 had missing and 30 had filled teeth. Twenty-five percent of subjects had undergone filling process and about 12% of subjects had missing teeth (Table 2). Malocclusion of class II div I type, class II div II type and class III was found in 20, 11 and five subjects respectively. Spacing in dentition was seen in 14.65% and rotation in 8.65% of population (Table 3). 

**Table 2:** Decayed, missing and filled teeth in children

Parameters	Number (n)	Percent (%)
Decayed	25	21.5
Missing	12	10.3
Filled	30	25.8

**Table 3:** Assessment of malocclusion

Malocclusion	Number (n)	Percent (%)
Class II div I	20	17.24
Class II div II	11	9.48
Class III	5	4.31
Spacing	17	14.65
Rotation	10	8.6

It was found that 32 had mild fluorosis and 74 had gingivitis. The percentage of mild fluorosis and gingivitis was 27.58% and 63.79% respectively (Table 4). The method of communication was sign language, lip-reading and hearing aids in 62%, 20% and 18% respectively (Table 5).

**Table 4:** Assessment of fluorosis and gingival diseases

Parameters	Number (n)	Percent (%)
Mild fluorosis	32	27.58
Gingivitis	74	63.79

**Table 5:** Method of communication

Communication method	Number (n)
Sign language	62%
Lip reading	20%
Hearing aids	18%

## Discussion

Oral hygiene of children is poor in contrast to adults. The reason can be lack of awareness, increased sugar intake, and poor dietary habits [10,11]. Oral health is an essential pattern of health for children, especially in children with special health needs [12,13]. It has an impact on social well-being [14,15]. Children with special care as those with hearing and speech disabilities need extra attention as they cannot maintain their oral hygiene as effectively as can be managed by normal children [16,17]. The present study was conducted to assess oral health status in hearing- and speech-impaired children in Jeddah city.

Our results showed that 16 children belonged to the age group of five to seven years, 25 in eight to 10 years, 30 in 11 to 13 years, and 45 in the age group of 14 to 16 years. Of 116 children, there were 60 males and 56 females. Kalaivani et al. enrolled 75 hearing- and speech-impaired children aged seven to 14 years of both sexes [18]. All were thoroughly examined. Out of 75, males were 46 (61%) and females were 29 (39%). Dental caries was a common dental disease found in 65% of children and gingival bleeding in 47% of children. It was observed that 76% of children required prompt treatment. Intervention urgency was highest among those children who have not visited a dentist before.

Our results demonstrated that among 116 children, 25 had decayed, 12 had missing and 30 had filled teeth. A study by Suma et al. in 2011 on 76 children with special care needs (speech and hearing impairment) comprised 47 males and 29 females [19]. 80.26% of them had never visited the dentist whereas 14.47% reported to the dental surgeon in case of pain. Common treatments done were extraction and restorations. It was seen that 71.05% of children felt that fizzy drinks and 56.58% of children that sweets have no adverse effect on teeth. About 71% found that brushing teeth prevent dental decay and > 90% of them cared about their teeth as much as any other part of their body. Only once the daily habit of brushing was seen among 82.89% and twice daily in 17.11%. 42.11% of them brushed for about two minutes and 55.26% of the children were advised by parents to brush properly [19].

In our study malocclusion was found to be class II div I in 20 children, class II div II in 11, class III in five, spacing in 17, and rotation in 10. It was found that 32 had mild fluorosis and 74 had gingivitis. Kumar et al. observed that periodontal health was generally poor in all the children [20]. Oredugba et al. in their study suggested that insufficient knowledge about good oral hygiene practices in school authorities, absence of motivation, the minimal priority given to oral healthcare in the society, and poor socioeconomic status (SES) of parents or guardians could have resulted in poor oral hygiene among the disabled children [21].

The limitation of the study was that the sample size was small. Further, the study should also include the relationship of dental caries with brushing frequency, eating habits, dental visits and obesity in children. The history-taking regarding these variables should also be included considering them as potential risk factors for poor dental health among children with hearing and speech impairment.

## Conclusions

Children with special care needs such as hearing and speech disabilities had poor oral hygiene status. A careful oral examination is needed in these children. Sufficient steps by Saudi Arabia’s dental, community, and public health authorities are to be done to tackle the difference in oral health and hygiene among disabled children and also address the barriers to oral care that include cost, fear, and social attitudes. With an appropriate plan, comprehensible communication, and vigilantly drawn limits to services provided, the vivid oral health negligence by so many disabled children can be effectively lessened.

## References

[1] Ubido J, Huntington J, Warburton D (2002). Inequalities in access to healthcare faced by women who are deaf. Health Soc Care Community.

[2] Nunn JH (1987). The dental health of mentally and physically handicapped children: a review of the literature. Community Dent Health.

[3] Nagaraja Rao G (1985). Oral health status of certified school children of Mysore state--a report. J Indian Dent Assoc.

[4] Gupta DP, Chowdhury R, Sarkar S (1993). Prevalence of dental caries in handicapped children of Calcutta. J Indian Soc Pedod Prev Dent.

[5] Bhavsar JP, Damle SG (1995). Dental caries and oral hygiene amongst 12-14 years old handicapped children of Bombay, India. J Indian Soc Pedod Prev Dent.

[6] Siddibhavi MB (2012). Oral health status of handicapped children attending various special schools in Belgaum city
Karnataka. WebmedCentral.

[7] Desai M, Messer LB, Calache H (2001). A study of the dental treatment needs of children with disabilities in Melbourne, Australia. Aust Dent J.

[8] Shyama M, Al-Mutawa SA, Morris RE, Sugathan T, Honkala E (2001). Dental caries experience of disabled children and young adults in Kuwait. Community Dent Health.

[9] Choi NK, Yang KH (2003). A study on the dental disease of the handicapped. J Dent Child (Chic).

[10] Mouradian WE (2001). The face of a child: children's oral health and dental education. J Dent Educ.

[11] Franks AS, Winter GB (1974). Management of the handicapped and chronic sick patient in the dental practice. Br Dent J.

[12] Modéer T, Wondimu B (2000). Periodontal diseases in children and adolescents. Dent Clin North Am.

[13] Purohit BM, Singh A (2012). Oral health status of 12-year-old children with disabilities and controls in Southern India. WHO South East Asia J Public Health.

[14] Jain M, Mathur A, Kumar S, Dagli RJ, Duraiswamy P, Kulkarni S (2008). Dentition status and treatment needs among children with impaired hearing attending a special school for the deaf and mute in Udaipur, India. J Oral Sci.

[15] Sanjay V, Shetty SM, Shetty RG, Managoli NA, Gugawad SC, Hitesh D (2014). Dental health status among sensory impaired and blind institutionalized children aged 6 to 20 years. J Int Oral Health.

[16] Vishnu P, Mahesh R, Madan Kumar PD, Sharna N (2021). Oral health status and treatment needs of children with sensory deficits in Chennai, India-a cross-sectional study. Indian J Dent Res.

[17] Aruna CN, Chandu GN, Shafiulla MD (2005). Dental caries experience among deaf and dumb children in Davengere, Karnataka. J Indian Assoc Public Health Dent.

[18] Kalaivani S, Shavi GR, Shanmugam S (2021). Oral health status of hearing and speech-impaired schoolchildren in Erode district, Tamil Nadu - a cross-sectional study. SRM J Res Dent Sci.

[19] Suma G, Das UM, Akshatha BS (2011). Dentition status and oral health practice among hearing and speech-impaired children: a cross-sectional study. Int J Clin Pediatr Dent.

[20] Kumar RH, Khandare AL, Brahmam GN (2007). Assessment of current status of fluorosis in North-Western districts of Tamil Nadu using community index for dental fluorosis. J Hum Ecol.

[21] Oredugba FA (2004). Oral health care knowledge and practices of a group of deaf adolescents in Lagos, Nigeria. J Public Health Dent.

